# Adapting an evidence-based contraceptive behavioural intervention delivered by mobile phone for young people in Zimbabwe

**DOI:** 10.1186/s12913-022-07501-9

**Published:** 2022-01-25

**Authors:** Ona L McCarthy, Constancia Mavodza, Chido Dziva Chikwari, Ethel Dauya, Mandikudza Tembo, Portia Hlabangana, Regedzai Dembetembe, Nyasha Mpakami, Tsitsi Bandason, Caroline Free, Chris Smith, Rashida A Ferrand

**Affiliations:** 1 London School of Hygiene and Tropical Medicine, Keppel Street, London, WC1E 7HT England; 2 Biomedical Research and Training Institute, 10 Seagrave, Avondale, Harare, Zimbabwe

**Keywords:** Adaptation, Digital intervention, Implementation, Evidence-based, Contraception, Young people

## Abstract

**Background:**

Despite the availability of a range of contraceptive methods, young people around the world still face barriers in accessing and using them. The use of digital technology for the delivery of health interventions has expanded rapidly. Intervention delivery by mobile phone can be a useful way to address young people’s needs with regard to sexual and reproductive health, because the information can be digested at a time of the recipients’ choosing. This study reports the adaptation of an evidence-based contraceptive behavioural intervention for young people in Zimbabwe.

**Methods:**

Focus group discussions and in depth interviews were used to evaluate the ‘fit’ of the existing intervention among young people in Harare, Zimbabwe. This involved determining how aligned the content of the existing intervention was to the knowledge and beliefs of young Zimbabweans plus identifying the most appropriate intervention deliver mode. The verbatim transcripts were analysed using a thematic analysis. The existing intervention was then adapted, tested and refined in subsequent focus group discussions and interviews with young people in Harare and Bulawayo.

**Results:**

Eleven key themes resulted from the discussions evaluating the fit of the intervention. While there were many similarities to the original study population, key differences were that young people in Zimbabwe had lower levels of personal and smart mobile phone ownership and lower literacy levels. Young people were enthusiastic about receiving information about side effects/side benefits of the methods. The iterative testing and refinement resulted in adapted intervention consisting of 97 messages for female recipients (94 for male), delivered over three months and offered in English, Shona and Ndebele.

**Conclusions:**

Young people in Zimbabwe provided essential information for adapting the existing intervention. There was great support for the adapted intervention among the young people who took part in this study. The adapted intervention is now being implemented within an integrated community-based sexual and reproductive health service in Zimbabwe.

**Supplementary Information:**

The online version contains supplementary material available at 10.1186/s12913-022-07501-9.

## Contributions to the literature

- Many digital health interventions are developed globally. Fewer are evaluated and those that are and show a positive effect on health outcomes are not always implemented.
- Adapting evidence-base interventions for different contexts is a financially and time efficient way to expediate the delivery of effective interventions to the people that need them.
- This intervention adaptation study demonstrates that intervention adaptation can be done rigorously and in a relatively short amount of time, despite unanticipated disruptions caused by the COVID-19 pandemic.

## Background

Complications from pregnancy and childbirth are the leading cause of death among 15–19 year old girls globally [1]. Access to comprehensive contraceptive information and family planning services can reduce adolescent pregnancies and births [1]. Yet young people face numerous barriers to accessing and using contraception- for which effective interventions are needed [2].

The global expansion of use of personal digital technology has offered new routes in which to deliver health interventions [3]. Mobile phones interventions can be particularly advantageous for addressing young people’s sexual and reproductive health (SRH) as they can be received in private, allowing the recipient to digest the information in their own time. The support can be non-judgemental, can reach many people and is often more convenient and cheaper to deliver than face-to-face support.

In 2015, a multidisciplinary team of researchers developed a contraceptive behavioural intervention in Tajikistan, the Occupied Palestinian Territories (oPT) and Bolivia, delivered by mobile phone to improve young people’s attitudes towards and use of effective contraception. The intervention is grounded in behavioural science [4] and consists of short mobile phone messages providing contraceptive support delivered over four months. Evaluation of the intervention demonstrated that it can improve knowledge and acceptability of and intention to use effective contraception [5, 6].

In 2019 researchers in Zimbabwe sought to adapt the existing mobile phone contraceptive behavioural intervention (hereafter referred to as ‘the existing intervention’). The adapted intervention would be added to an integrated community-based package of HIV and SRH services for 16–24-year olds in three provinces in Zimbabwe (‘CHIEDZA’), which is being evaluated in a cluster randomised controlled trial (ClinicalTrials.gov Identifier: NCT03719521). The benefits of integrating HIV and SRH are widely recognized and include improved access to and uptake of both HIV and SRH services [7]. The rationale for adding a mobile phone components to the CHIEDZA package was to enhance the integration of its reproductive health and HIV services, by offering young people the opportunity to receive information about contraception in their own space, extending the CHIEDZA package beyond its face to face offering.

This paper describes the process and results of adapting the existing intervention to the Zimbabwean context, with the aim of integrating it within the CHIEDZA intervention package.

## Methods

The overall aim of the project was to produce an adapted intervention, that accounts for the needs and preferences of young people, that is clearly described and maintains the core elements of the original intervention. The adaptation approach drew upon elements of the Intervention Mapping adaptation framework (IM Adapt) [8]. All researchers were trained in data collection using qualitative methods and had prior experience in conducting focus group discussions (FGDs) and in-depth interviews (IDIs). FGDs ad IDIs lasted up to one hour.

### Evaluating the fit of the existing intervention

To evaluate the fit of the intervention in the Zimbabwean context and to identify the best delivery mode, we conducted FGDs and IDIs with men and women aged 16–24 in two CHIEDZA intervention cluster services in one of the provinces in which the trial is being implemented (Mashonaland East) from October 2019. Specific objectives of the FGDs and IDIs were to determine how aligned the existing intervention was with the knowledge and beliefs of young Zimbabweans regarding contraception, including understanding their preferences for intervention delivery. An additional objective was to explore their HIV risk perception with a view to adding safer sex content to the existing intervention.

One female and one male researcher (RD and NM) facilitated the FGDs and IDIs. The discussion guide (Additional File 1) mirrored the structure of the existing intervention. The approach was to assess how similar young Zimbabweans’ beliefs about contraception were to the contexts where the intervention was first developed; differences would be reflected in the adapted intervention. Mashonaland East was selected because of its proximity to Harare, where the facilitators were based. All CHIEDZA service users were eligible to take part (i.e. men and women aged 16–24) and participants were identified and recruited from CHIEDZA services by the facilitators via convenience sampling. The FGDs and IDIs were held in the Shona language and audio recorded. An ‘orthographic’ transcript, i.e. a verbatim account of all that is said plus non-verbal utterances [9] was transcribed and translated into English.

### Analysis of the initial FGDs and IDIs

The analysis of the initial FGDs and IDIs followed Braun and Clarke’s (2006) guide to conducting a thematic analysis [9]. One coder (OM) analysed the data using a theoretical thematic analysis, driven by the analytical interest in the topic (adapting the existing intervention), coded for the specific research question. The first step was familiarisation with the data set by rereading the transcripts to find patterns of meaning. Next, initial codes were generated after listing ideas about what the data contains. Codes were identified at the semantic level and then the data was organised into meaningful groups. The coding was theory-driven, i.e., there were specific questions to code on. The coding was completed using word processing with no specific coding software. Codes were assigned using comments boxes. The assumption that informed the analysis was that young Zimbabweans’ beliefs would be similar to the beliefs identified among young people in Tajikistan, Bolivia and the oPT [4]. Data was sought to answer the research question, which was driven by the theoretical framework of the existing intervention. The next step was to search for themes, which were the units of analysis. This was done by collating the codes from the long list across the data set into potential themes. Then, all the data was gathered (the coded extracts) under each potential theme. The themes were reviewed in relation to the research question, to identify key themes that would be relevant for the intervention adaptation. The themes were also checked in relation to the coded extracts to make sure they made sense. Finally, a clear definition and name was generated for each theme. All the themes and data underneath them were reviewed again, summarised and refined.

### Desk-based adaptation

The results of the analysis of the initial FGDs and IDIs were then used to produce a first draft of an adapted intervention to test in the testing and refining phase. This involved comparing the themes to the results of the intervention development of the existing intervention, for example, comparing the results to the existing logic model of the problem.

### Testing and refining the content

The aim of this stage was to refine the intervention content so that it is relevant and culturally appropriate. Study researchers completed the FGDs and IDIs in rounds (completed by CM, CDC and PH), with the intervention content refined (by OM, CM and CDC) after each round based on key feedback from the participants. The discussion guide was also updated after each round, for use in the next round (Additional files 2, 3 and 4). The final intervention was produced by the core research team (RAF, OM, CM, CDC and ED) after a review of the findings from the testing stage.

## Results

### Evaluating the fit of the existing intervention

Four FGDs and four IDIs were conducted in this stage with 39 participants (18 female and 21 male). Eighty-three initial codes were generated, which were grouped into 17 potential themes. Reviewing and only retaining themes directly relevant to adapting the intervention resulted in 11 final key themes:

- **Theme 1: Personal mobile phone ownership and smart phone ownership is not universal**. Many participants reported that they did not own a personal mobile phone but most had access to one (e.g. they shared a family member’s). Some participants did not have access to a ‘smart phone’ (a mobile phone that has advance technical functionality such as Internet access, data storage and email capability).
- **Theme 2: Contraception information delivered to your mobile phone is convenient**. Participants spoke about the convenience of receiving contraceptive information on the phone; young people sometimes do not have time to visit the clinic and receiving information on their phone means that they do not have to travel far distances to the clinic for it. They talked about the ‘stress of walking long journeys’ to the clinic and the 'burden of travelling'. Other conveniences mentioned were that the information can be shared with others and read at a time of their choosing. There was a lot of enthusiasm for learning and gaining knowledge through SMS—participants appreciated that receiving knowledge through this mode is a good way to learn. One participant was enthusiastic about reading the information on the phone because they would not have to deal with feeling 'shy' with a face-to-face discussion with an adult. Others spoke about being 'afraid' and 'ashamed' to ask adults about contraception face-to-face and noted that receiving information on their phone is a convenient way around this.
  *“It is a good idea to receive this information on your phone because you are able to read it on your own at your convenience “* (Theme 2, FGD-01-male)

- **Theme 3: SMS is the most accessible delivery mode.** There was almost universal preference for SMS as the mobile intervention delivery mode, which was primarily based on financial factors. Participants preferred SMS because they are cheap—receiving messages through WhatsApp (the predominant instant-messaging application in Zimbabwe) requires the user to purchase data bundles, whereas SMS can be received anytime. In addition, participants sometimes they did not have access to a phone that can support WhatsApp.
  *“Text messages are more appropriate since every cell phone has access to text messages and also it is easy to get information as compared to WhatsApp and internet where one has to buy data to have access to the messages.”* (Theme 3, IDI-02-male)

- **Theme 4: Young people have varying levels of knowledge about contraception.** The implant, intra-uterine device (IUD ‘loop’), oral contraceptives, injection, emergency contraception and condoms were widely known among participants. However, few participants seemed to have a deeper knowledge about how the methods are actually used or how they work.
  *“I don’t understand much, all l know is that it’s inserted in the uterus.”* (Theme 4, IDI-02-female)

- **Theme 5: Young people hold negative views about contraception.** Participants expressed a range of negative views about contraception. The most common concern was contraception’s perceived effect on fertility after discontinuation, with participants specifically mentioning the implant (referred to by the brand name commonly available, ‘Jadelle’), oral contraceptives and injections as causing infertility or problems with conceiving. Participants felt that condoms were better because they do not affect fertility and that long acting methods were not good for the newly married and more appropriate for after childbearing. Participants also expressed concerns regarding the side effects of the implant, particularly prolonged bleeding, causing men to ‘run away’. Some participants heard that men can feel the IUD and that it ‘stores dirt’ and causes it to accumulate in the uterus. There was further anxiety around the expiration date of Jadelle, with participants expressing uncertainty around how long it is effective for. A Jadelle inserted that is ‘about to expire’ could cause an unintended pregnancy and could prevent a planned pregnancy if you get married before it has expired. One participant had heard that contraception could cause uterine cancer. Another view was that if women used emergency contraception frequently, it would become ineffective.

“*For boys its common for them to use condoms but it’s not common for young girls to be using contraceptives because we were told that it is not good for girls to take contraceptives before they have children it will affect their fertility.”* (Theme 5, FGD-01-male)

- **Theme 6: Young people use and value condoms but are not confident using them.** Participants spoke about how common condom use is among young people, however some are ‘shy’ to purchase them. They also spoke about how they lacked confidence in using them successfully, saying that they needed more knowledge about how to use them without having them burst. Participants talked about how condom use within marriage could cause relationship conflicts. One male participant mentioned that some women do not agree with using condoms with their partners because they feel that condoms are meant for sex workers. There were no major differences in views between male and female participants.
  *“Most youths use condoms but they do not have enough knowledge in terms of how to wear the condoms. They do not know the proper way of putting it on and in most cases its done in rush.”* (Theme 6, FGD-02-male)

- **Theme 7: It is very easy to become infected with HIV though sex without a condom.** Sex without a condom was viewed as very risky with regard to HIV infection. Many participants also mentioned not knowing their partners’ status.
  *“It is very easy because if my partner has STIs and am not aware of it, so they are high chances of me getting HIV.”* (Theme 7, FGD-01-female)

- **Theme 8: Joint contraception decision making, communication, and use is difficult within marriage.** Participants spoke about how married women are valued for being a mother, and society and the wider family expect this role will follow shortly after marriage. Because contraceptive use within marriage revolves around whether the couple already have a child, participants thought that joint decision making is not an accepted concept and use is difficult. A male interviewee also mentioned that it is not acceptable for women to use contraception if they have not given birth.
  “*I don’t give my own decisions. My husband does.”* (Theme 8, FGD-02-female)
  *“That’s difficult, because, when we get married we want to have a family, and when the woman refuses to have children, the man wonders, why he got married. That is when a husband will make a decision to have a small house (he chuckles),”* (Theme 8, IDI-01-male)

- **Theme 9: Confidence in talking about contraception varies, but most do not feel confident talking to partners or providers.** Young people mentioned that some women are afraid to talk to their husbands about contraception, particularly if they are a lot younger, and that the husband has the authority to make decisions about contraception. Some young male participants, however, thought that young men and women are confident talking to partners. Others thought it depends on how close you are with your partner. In clinics, young people do not have confidence talking to providers because they think that providers will tell them that they are too young to use contraception (if they are under 18). Participants mentioned choosing to collect condoms at the clinic when no one is watching, out of fear that clinic staff will think negatively of them (primarily among unmarried young people).
  *“If we ask, they* [providers] *will tell us that you have not reached the age of getting contraceptives which 18 years not 16 years hence by this it reduces our confidence level to ask anything.“* (Theme 9, FGD-01-female)
  *“I don’t even have that confidence even to get the condom from my pocket to use it, it’s difficult to talk to your partner because you don’t know how she will react to it.”* (Theme 9, FGD-02-male)

- **Theme 10: Young women fear trying new methods.** Young women spoke about the ‘fear’ around how their body would respond to a contraceptive method they have not used before. They thought that hearing about other women’s experiences in trying contraceptive methods they have never tried before would help them feel more confident.
  *“It’s scary because I want to know whether it’s going to be accepted by my body or might get side effects so it’s scary to change”* (Theme 10, FGD-02-female)

- **Theme 11: Preferences for intervention content.** Regarding intervention content, participants wanted information on condom use and contraception, ‘testimonies’, how contraception works and side effects/benefits of contraception. They preferred one to three short messages a day offered in Shona and English.
  *“I want to receive everything.”* (Theme 11, FGD-02-female)
  *“I think at least three messages, maybe in the morning, in the afternoon and in the evening.”* (Theme 11, IDI-01-male)

### Desk-based adaptation

The content of the existing intervention was largely aligned with the knowledge and beliefs of the young Zimbabweans that participated in the discussions. Some differences were expressed; fear of trying new methods, a high level of HIV risk perception and lack of confidence using condoms successfully and a lower level of personal and smart phone ownership.

### Similarities to the existing intervention

As with the previous study population (in Tajikistan, Bolivia and the oPT), participants wanted information on side effects/side benefits and testimonies in one to three short messages a day in multiple languages. Although young people in Zimbabwe expressed greater confidence about talking to their partners about contraception compared to the previous study population, this varied and most were not confident talking to partners or providers. Participants expressed that there was a strong cultural importance placed on the role of the mother and the familial and societal expectation that a pregnancy will soon follow marriage. Participants expressed many of the same negative beliefs about contraception that the previous study population expressed. While participants generally had a greater level of knowledge about contraception compared to the previous study population, knowledge levels ranged and there were some participants with very low levels of knowledge. As with the previous study population, young people were very enthusiastic about receiving information about contraception on their mobile phone.

### Differences from the existing intervention

There was more expressed fear about trying contraceptive methods for the first time among this group of Zimbabwean young women compared to the previous study population. Susceptibility to HIV infection through sex without a condom and condom use were additional topics added in this adapted intervention, which were not included in the existing intervention. Of the three countries where the intervention was originally developed, only in the oPT was SMS the widely preferred intervention delivery choice. Similar to young people in the oPT, young Zimbabweans in this current study thought that SMS was the best option because not everyone had access to phones with WhatsApp and Internet functionality and if they did, they spoke of the barrier of having to buy data ‘bundles’ to support the cost of receiving the WhatsApp messages. There were lower levels of personal mobile phone and smart phone ownership among participants compared to the original study population.

**Fig. 1:**
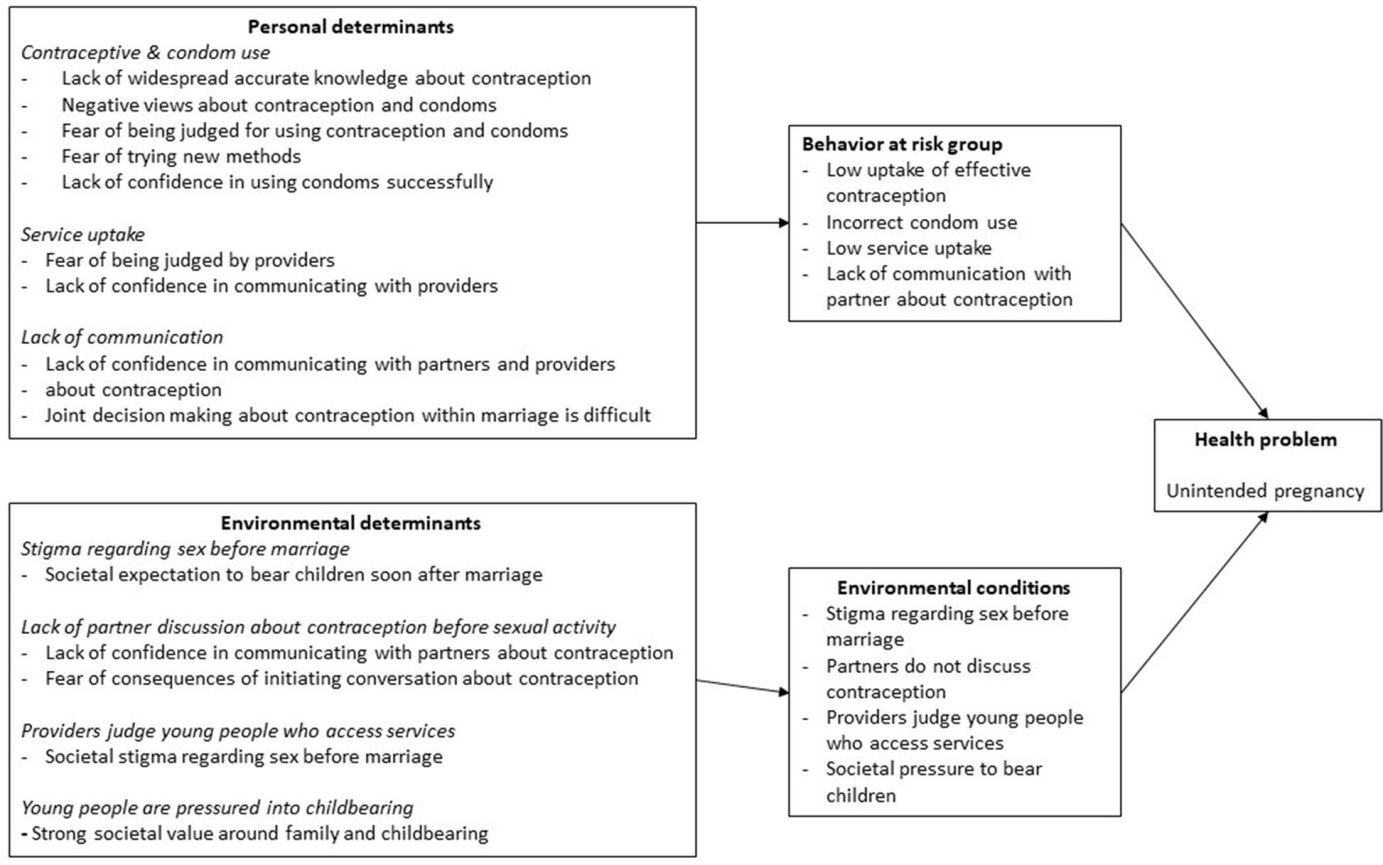
Logic model of the problem

Figure 1 presents the logic model of the problem of the existing intervention [4] updated to encompass the themes from the focus group discussions and interviews in this current Zimbabwean study.

### Summary*** of adaptations***

This first round of adaptations was very conservative regarding removing messages so as to maintain the core components of the existing intervention as much as possible; messages were only removed if it was clear that they were not relevant, based on what was known at the time about the context and what was known about young people’s preferences from the FGDs and IDIs.

Messages regarding correct condom use (e.g. how to use condoms so they are less likely to break), taken from an existing SMS intervention [10] were added. Three messages for non-sexually active people were condensed into one message in the main adapted Zimbabwean message set to account for periods of sexually active and inactivity (for both the male and female sets). Some messages were combined, where it made sense to do so and was simple, to reduce the total number of messages. Some content was updated to reflect the Zimbabwean context (e.g. the patch was not available at the time). Terms commonly used in Zimbabwe for the different methods were changed (e.g. the ‘control pill’ for the combined pill and the ‘secure pill’ for the progesterone only pill). Some messages in the existing intervention that addressed beliefs that were not raised in the FGDs and IDIs were retained because they are common in the global literature (e.g. concerns about weight gain and hormones). The concerns expressed about contraception were more around long-term methods rather than hormonal aspect of them. These were retained because it was not completely clear what the concern was. The term ‘family planning’ was changed to ‘contraception’ throughout. The terms ‘wife’ and ‘husband’ were changed to ‘partner’ throughout because sex before marriage was frequently referred to. Finally, the messages suggesting that people could attend a service further away from their neighbourhood were removed because participants spoke about the burden of having to travel far distances; advising them to travel further would not be relevant and would decrease the credibility of the intervention.

### Testing and refining the content

Four FGDs and six IDIs were originally planned in this phase, but the emergence of the COVID-19 pandemic necessitated a switch to conducting only IDIs for a while because it was easier to maintain physical distancing compared to FGDs. This resulted in 10 IDIs and two FGDs, conducted over three rounds of testing.

#### Round 1

The first round of testing involved four interviews (one female and one male in Harare; one male and one female in Bulawayo). Interviewees were given the messages in paper form and were asked to share their overall impression of the messages, the tone and content. All participants were very positive about the prospect of receiving the messages on their phone. They wanted the messages to be sent to young men as well as women and liked the variety of the messages (the combination of contraceptive and safer sex content). They thought the messages about how to initiate a conversation about contraception with a partner were helpful as it would ‘break the ice’. Interviewees suggested that young people should opt in to receive the messages and not receive them without requesting them (this was the original plan).

Round one participants had some suggestions for improvement. They suggested that we do not use abbreviations on their own. They found some of the language too technical. One participant commented that there was some repetition, but went on to say that if it was for emphasis, then that was ok. A participant wanted more information about why there are so many contraceptive methods that work differently and if there are methods besides condoms for men. They wanted the myth about contraception and the fact in the same message and to specify the exact service that CHIEDZA offers.

After a team discussion we refined the intervention accordingly: abbreviations were accompanied with the entire phrase (with the exception of sexually transmitted infections, where ‘STIs’ is what is commonly used- the first time STIs are mentioned, it is spelled out in full with the abbreviation); the language was simplified so that the meaning is clearer (we also added questions about informal language to the Round 2 testing discussion guide). We retained potentially repetitious messages because they are intended to be delivered over a period of time, rather than in one sitting as was done in this round of testing, which would have made them seem repetitious. We altered a message to say that there are many available methods to provide choice about how contraception is taken. Regarding the fact and myth messages, we scheduled these to be delivered on the same day.

*“What is the reason for having many contraceptive methods which work differently? I really want an explanation on that to understand why the methods are many but work differently… Isn’t there another method that we can use other than condoms?” (Round 1-IDI-male-Harare)*

“As for me I feel great opening such messages and if you are to send the messages to me I will open them freely because I want to learn.” (Round 1-IDI-male-Harare)

#### Round*** 2***

In Round 2, we conducted six interviews (one female in Bulawayo and two females and three males in Harare). We intended to conduct four interviews, but due to literacy barriers among some of the young people we approached in this round, we conducted an additional two. In this round, we focused on understanding what participants’ views were on the tone of the messages and how well they understood specific content and terminology. During this period, two young people were trained in FGD facilitation (one young woman in Harare and one young woman in Bulawayo) and co-facilitated one FGD each.

There were mixed views regarding the use of informal language in the messages; some thought that ‘slang’ would aid comprehension and engagement among young people and another view was that Shona or Ndebele is better because slang varies from area to area. All participants thought would be happy to receive the messages. They appreciated the messages about talking to a partner about contraception and the messages that address myths and thought that the tone was supportive. Participants offered suggestions for how to improve specific messages, so that they would not require an ‘additional person’ to help them understand the meaning.

*“I think the messages were coming from someone who really wants us as the youth to know about pregnancy and contraceptives since this is something good. So wherever this person is, they really did a great job in writing the messages and trying to make us young people understand our health systems and take care of ourselves in the process as well”* (Round 2-IDI-male-Harare)

*“Well I think it quite useful to have such knowledge, but maybe the challenge might come when one is dealing with a partner who does not want to discuss such matters so it might be difficult to talk to my partner about the issue of contraceptives. However, the information is very useful especially when you get one whose willing to talk about such matters.”* (Round 2-IDI-female-Bulawayo)

In response to what participants reported in Round 2, the team decided not to use slang because would be too difficult to ensure that content could be understood by all. To address the low literacy levels, we further simplified the language and separated some of the longer messages. We adjusted messages to improve the likelihood of comprehension, e.g. we changed ‘The pill does not cause infertility’ to ‘Women can become pregnant soon after stopping the pill’.

#### Round 3

The final round of testing involved two FGDs- one female group with seven participants in Harare and a mixed female and male group with 10 participants (five female and five male) in Bulawayo. While the messages were largely acceptable, participants offered suggestions for improvement. There were mixed views about the use of slang as in Round 2. Some suggested sending the same message in different languages. Two participants raised the issue of literacy, asking how we will accommodate for people who have lower literacy levels, suggesting that we also send the messages as pictures. However, others acknowledge that not all young people have smartphones. One participant suggested that the intervention contain more ‘dialogue’- conversations between males and females in ‘real situations faced by real people not just mere messages’.

*“Maybe the messages can be sent at intervals for instance saying that at 10 a.m a Shona message is sent then at 11 a.m an English message is sent. So someone will get to choose which message they want to read so 2 messages mixed with both Shona and English will do”* (Round 3-FGD-female-Harare)

*“I want to tell the truth honestly if I see a message that says something health related and with a salutation signature written “CHIEDZA” I will definitely read it because I will also remember that it is the same CHIEDZA that assisted me with free pads and condoms so yes definitely if it comes from CHIEDZA I will read it .” (Round 3-FGD-female-Harare)*

After Round 3, the wider team reviewed the messages and made final refinements.

### Description of the final adapted intervention

The final adapted intervention consisted of 97 messages in the female set (Additional File 5) and 94 in the male set, delivered over three months and offered in English, Shona and Ndebele. The majority of the messages were similar between the two sets, with minor tailoring for gender. The messages cover the same content as the original intervention, with the addition of condom use messages. The messages provide accurate information about contraception and condom use and contain the following behaviour change methods [11] from the original intervention [4] adapted for delivery by mobile phone: verbal persuasion; tailoring; guided practice; cultural similarity; belief selection; arguments and anticipated regret. (The behaviour change method *cultural similarity* was in the form of quotes, which were derived from young people in the oPT in the development of the original intervention, checked for applicability in Zimbabwe.)

## Discussion

### Main findings

We adapted an existing contraceptive behavioural intervention to account for the needs and preferences of young people in Zimbabwe. The adapted intervention is clearly described and maintains the core elements of the existing intervention. Compared to the original study populations, young people had lower levels of personal and smart mobile phone ownership and lower literacy levels.

### Comparisons with existing research

Recent research has suggested that mobile phone interventions may be feasible among young people in Zimbabwe but have the potential to increase inequities if internet access is a prerequisite for intervention receipt [12]. The current study was in line with this finding, with young people favouring a non-internet dependent intervention. Mobile internet access may improve over time, and it would then be possible to further develop the intervention to include a greater array of interactivity. However, mitigation of the potential inequities that such digital health innovations can create or worsen should always remain an essential component of intervention development and implementation [13].

### Limitations

This project has several methodological limitations. Analysis of the FGDs and IDIs was completed by one coder, who led the development of the existing intervention. While the analysis was driven by the need to code on pre-specified questions, involvement in the development of the existing intervention could have influenced the coder’s interpretation of the data and decision making. This work was started a few months before the global COVID-19 pandemic and was conducted throughout. A potential impact of the pandemic could relate to the sample. Young people who were more willing to attend CHIEDZA services during the pandemic may have had different views about the intervention content compared to those who did not feel comfortable attending the service. This could have resulted in the intervention not reflecting the needs of a wider range of young people. However, it is reassuring that during the pandemic, CHIEDZA service use only slightly decreased. The topic guides were very structured to answer the research questions, which meant that there could have been many other themes that would have emerged had the intervention been developed from scratch. In general, it is a more efficient use of resources to adapt an appropriate existing evidence-based intervention.

### Implications

Many effective interventions fail to reach the population that can benefit from them, due in large part to inadequate knowledge translation of research findings [14]. Mobile phone-based interventions in particular are often piloted but lack a sufficient evidence base to inform implementation [15]. The adapted intervention was integrated within the CHIEDZA intervention communities in January 2021 using an ‘opt-in’ approach; clients are offered the intervention when they attend the service. This adaptation work drew upon an established intervention adaptation approach [8] and was guided by an established qualitative analytic method [9]. As the adapted intervention maintains the key components of the existing intervention, efforts to evaluate the adapted intervention will be relatively straightforward as the active components are defined. Future development of the adapted intervention should focus on increasing its accessibility, i.e. to those without personal mobile phones, mobile Internet access and to those with lower literacy levels. For example, offering audio and visual content on mobile phones and ensuring that the information is delivered in an alternative mode, where the phone is an adjunct to care.

## Conclusion

The young people in Zimbabwe with whom we spoke enthusiastically supported the idea of receiving information about contraception on their mobile phone. They provided essential information to enable the adaptation of the existing intervention. The adapted intervention has been implemented, and there is a need to evaluate implementation and its effectiveness on contraceptive uptake among CHIEDZA service users.

## Availability of data and other materials

The datasets generated during and/or analysed during the current study are available from the corresponding author on reasonable request.

## Supplementary Information

**Additional file 1. **

**Additional file 2. **

**Additional file 3. **

**Additional file 4. **

**Additional file 5. **

**Additional file 6. **

## Abbreviations

oPT: Occupied Palestinian Territories
HIV: Human immunodeficiency virus
SRH: Sexual and reproductive health
FGD: Focus group discussion
IDI: In-depth interview
