# Supplementary material for: Adapting an evidence-based contraceptive behavioural intervention delivered by mobile phone for young people in Zimbabwe
Source: BMC Health Serv Res. 2022 Jan 25;22:106. doi: 10.1186/s12913-022-07501-9 (PMC8789333; doi:10.1186/s12913-022-07501-9)
Supplement: Supplementary file 1 — Additional file 1. [file 12913_2022_7501_MOESM1_ESM.pdf]

v1 22 October 2019

Discussion guide used to evaluate the fit of the existing intervention

CHIEDZA formative work discussion guide

1. Introduction

- We would like to hear about your experiences and thoughts about contraception
- And about what you think of receiving contraceptive support by mobile phone
- Don't have to take part if you don't want to
- There are no right or wrong answers
- I am here to facilitate the discussion, I'm not an expert on the issues
- We are interested in a range of views, so it's ok to disagree with each other
- Check again that they are ok with audio recording, explain confidentiality and anonymity
- It will last up to 60 mins
- You can leave when you want without having to give a reason
- You don't have to talk about anything you don't want to talk about

2. Informed consent

- Check that they have all had time to read the information sheet
- Answer any questions
- Give them the consent form to sign (signed by you too)
- Drop off consent straight after at BRTI

Rules (for focus group):

- Please respect each other's confidentiality by not sharing anything with anyone outside the group that might identify people in the group
- Respect each other's opinions
- Don't interrupt
- Please one person speak at a time so that we can understand the recording
- Can take phone calls (please leave the room for the conversation)

START RECORDER

3. Technology & information

Aim: to understand patterns of technology use

- **Do you own a phone? If so, what kind? Do you access the internet on your phone? Social media (what kind?)? SMS?**
- Do you have a personal phone? If no, how do access cell phone service?
- **What is your opinion about receiving contraceptive information on your mobile phone? [Probe: why? elaborate, etc.]**
- How would you like to receive contraceptive support and information on your phone? [probe, social media, SMS, app]

4. Knowledge

Aim: to find out how much they know about the range of methods so we can identify any gaps in knowledge

- What kinds of contraception do young people know about?
- What do you know about them?
- How do young people find out about contraception?
- What does 'safer sex' mean to you?
- Would young people like more information about contraception in general? [how?]
- How easy is it for someone to become infected with HIV from unprotected sex?

5. Attitudes

Aim: to identify misconceptions, biases, perceptions and stigma surrounding contraception.

- What is the general perception about using contraception and condoms? [Why do you think this? Note: please separate contraception and condoms]
- What do *you* think about young people using contraception and condoms? [Do you know anyone who use them? What kinds do they use? Please separate contraception and condoms)
- At what age do you think it is appropriate for women to start using contraception? [Why?]
- What concerns (if any) do you have about these different methods? [if yes- which ones? Why do you think this?]
- Who should take responsibility for contraception? [you, your partner or both?]
- What do you think about unmarried young people having sex? [Why? Different for females & males?]

6. Barriers

Aim: to explore things that may prevent them from using contraception.

- How common it is for young people to use contraception and condoms? [ones have you/they used?]
- What kinds of problems (if any) do you or other people have with using contraception and condoms?
- A young woman in her teens just got married. How acceptable is it for her to wait to get pregnant?
- What role does religion play in how acceptable contraception is? [does this influence how you feel about it?]
- What do you think about women making decisions on their own about contraception? [how easy/hard is it for them? Why do you think this?]
- (F) If your partner didn't approve of contraception, would you use it anyway? [how important is it that your partner approves?]

7. Communication

Aim: to understand how confident they are talking to partners and others about their reproductive needs and preferences.

- How confident are young people with talking to their partner about contraception? [what would help them feel more confident?]
- How do young people feel about talking to staff at the clinic about contraception? [F- are young women comfortable talking to them on their own? Why/why not?]
- How confident are young people with talking to their friends and parents about contraception? (please separate a and b)
- If a young woman told her partner that she wanted to use contraception but her (male) partner said he didn't want to, what would happen?

8. Trying new methods

Aim: to get a sense for how open they are to trying new methods and how confident they feel about trying them.

- (F) If a young person wanted to try a new method, how confident would they be about trying it? [how easy/difficult would it be? What would prevent you from trying it? What would help young people feel more confident trying new methods?]
- What kinds of contraception do you think young people would be interested in trying? [Why]

9. Intervention content & privacy

Aim: to find out how the intervention can be supportive & easy to understand.

- What kinds of content should we send? [e.g. educational, true stories and quotes from other people about using contraception, ideas about how to talk about contraception with your partner, images, etc.]
- What kinds of content should we not send?
- What concerns would young people have about receiving content like this on your mobile phone?
- If the intervention was sent by short messages, how many messages should we send each day (if it was delivered by text or instant message)?

10. Wrap up

- Thinking about all that we talked about today, what do you feel is the most important? [why?]
- Any questions or comments? Thank you! (if they want any more information, show our contact details again)
