# Supplementary material for: Adapting an evidence-based contraceptive behavioural intervention delivered by mobile phone for young people in Zimbabwe
Source: BMC Health Serv Res. 2022 Jan 25;22:106. doi: 10.1186/s12913-022-07501-9 (PMC8789333; doi:10.1186/s12913-022-07501-9)
Supplement: Supplementary file 2 — Additional file 2. [file 12913_2022_7501_MOESM2_ESM.pdf]

v1 26 May 2020

Discussion guide used in Round 1 testing of the adapted intervention

CHIEDZA contraception study

co-design/message-testing discussion guide

INTRODUCTION

- *"We have developed mobile phone messages about contraception and safer sex, which young people who attend CHIEDZA can sign up to receive on their phone*
- *We talked to young people in Zimbabwe about contraception and safer sex. We used what we learned from these discussions to make the messages.*
- *Right now, we want to ask young people what they think of the messages, to make sure people like them*
- *Please be honest- it is important that we know what doesn't sound right so we can improve the messages"*

Key points to reiterate:

- Don't have to take part if you don't want to
- There are no right or wrong answers
- I am here to facilitate the discussion, not to answer any clinical questions or provide advice
- (*FGD*) We are interested in a range of views, so it's ok to disagree with each other
- Check again that they are ok with audio recording, explain confidentiality and anonymity
- It will last up to 60 mins
- You can leave when you want without having to give a reason
- You don't have to talk about anything you don't want to talk about

INFORMED CONSENT

- Check that they have all had time to read the information sheet
- Answer any questions
- Give them the consent form to sign (signed by you too)
- Drop off consent straight after at BRTI

Rules (for focus group):

- Please respect each other's confidentiality by not sharing anything with anyone outside the group that might identify people in the group
- Respect each other's opinions
- Don't interrupt
- Please one person speak at a time so that we can understand the recording
- Can take phone calls (please leave the room for the conversation)

PART 1: FIRST IMPRESSIONS OF THIS MESSAGES

- *"Here are the messages. We will send 0-3 messages per day, over 4 months (they will not all come at once)*
- *Please take 15 minutes to read through them.*
- *Please make any comments in the space to the right about the message. For example, if you do not understand any words or if you like or do not like anything about the message, please note this and why.*
- *After you read through the message, we will talk through any comments you had.*
- *Don't worry about reading the messages carefully. We are just interested in your first impressions.*
- *Also, this is not a test- we need young people to help us make the messages better"*

(After giving them 15 minutes to read through the messages)

START RECORDER

1. What is your overall impression of the messages? [EXAMPLE PROBES: How interesting were they?]

2. How do you think you would feel receiving them on your phone (0-3 messages a day over 4 months)?

3. What comments did you have on specific messages? [PROBE: if they say they liked/did not like any messages, really try to understand what it was that they liked/did not liken and why.]

PART 2: TONE

4. Who does it sound like the messages are coming from? Is this good? Why/why not? [PROBE: For example, CHIEDZA, from a clinician, a teacher, a friend, an aunt, etc.?]

5. How do the messages make you feel? [PROBE: For example, were you interested, confused, bored, relieved, excited? Sometimes the way messages 'come across' can make people more of less willing to read them.]

6. For example, what do you think about how #82 sounds?

   Remember it's about your health and you have the right to choose what is right for you regardless of how others think and feel.

7. How can we make the tone of the messages better so that more young people will like them and read them?

PART 3: CONTENT

8. Are there any messages that are not needed?
   If YES, which messages are not needed and why?
   If NO, clarify that they think that all the messages are needed and ask them why they think this.

9. Is there anything missing? (If yes, why should we include it?)

10. What do you think about messages #105-108? [PROBE: Are they useful? Why/why not?]

11. What do you think about the condom messages, #13-18? [PROBE: Are they clear? Are they useful? Why/why not?]

PART 4: NEW QUOTES & TRAINING

12. Do you have any experiences with contraception that we can use as an anonymous quote?
    If YES, ask them about their experience (the aim is to obtain a short, real quotes about experiences using each contraceptive method)

13. Would you be interested in being trained to conduct these interviews yourself, along with me? [*We want to help young people gain skills in collecting interview data, if they are interested. This would involve a short training with a researcher, a practice interview and then you would conduct an interview yourself, along with me*]

"Thank you very much! You have been so helpful. Please contact X if you would like more information about the study"
