# Supplementary material for: Adapting an evidence-based contraceptive behavioural intervention delivered by mobile phone for young people in Zimbabwe
Source: BMC Health Serv Res. 2022 Jan 25;22:106. doi: 10.1186/s12913-022-07501-9 (PMC8789333; doi:10.1186/s12913-022-07501-9)
Supplement: Supplementary file 5 — Additional file 5. [file 12913_2022_7501_MOESM5_ESM.pdf]

**The female intervention messages that were adapted in this study, in English**

| Day | Order | Message |
|---|---|---|
| 1 | 1 | Within the coming 3 months we'll send 0-3 messages daily about contraceptive methods and safe sex. |
| 1 | 2 | If you aren't sexually active now, the information will help if you be prepared for the future. |
| 2 | 3 | The messages were written by health researchers with input from young people in Zimbabwe. |
| 3 | 4 | If you feel unsafe because someone read the messages, you can call X |
| 4 | 5 | To stop the messages… |
| 5 | 6 | There are many contraceptive methods that are taken in different ways. This is to give people choice about what works best for them. |
| 6 | 7 | The methods that are the best at preventing pregnancy are: pills, the intrauterine device ('loop', IUD), implant (e.g. Jadelle) injection (e.g. Depo). |
| 7 | 8 | If used correctly, it is very unlikely that someone would get pregnant using pills, the intrauterine device ('loop', IUD), implant (e.g. Jadelle) injection (e.g. Depo). |
| 7 | 9 | These methods are safe. They all work by preventing a male's sperm from joining a female's egg (called 'fertilization'). |
| 7 | 10 | They are good at preventing pregnancy but do not protect from sexually transmitted infections (STIs). Condoms are the best way to protect against infections. |
| 8 | 11 | Using contraception and condoms together protects against pregnancy and infections. |
| 9 | 12 | Having condoms with you makes it more likely you'll use one. Find a time to put a few in your bag or pocket. You could also keep a supply in places where you have sex. |
| 10 | 13 | A condom may burst if there is air trapped inside. To prevent this, hold the tip of the condom between your forefinger and thumb and roll it down, making sure there are no air bubbles. |

| | | |
|---|---|---|
| 11 | 14 | To avoid the condom falling off after sex, while the penis is still hard, hold the condom in place while withdrawing the penis. |
| 12 | 15 | Another reason a condom could burst is because it ripped when you opened the packet. To prevent this, before you open the packet, feel for the rim of the condom and push it aside, making sure you don’t tear the condom when you open the packet. |
| 13 | 16 | It could also split if the condom is out of date. Make sure to check this. |
| 14 | 17 | You can also use water or silicone-based lube with condoms. But don’t use anything oil-based (like Vaseline) because they can make the condom break. |
| 16 | 18 | Over the next few weeks, we will send you messages about pills, the intrauterine device ('loop', IUD), implant (e.g. Jadelle) and the injection (e.g. Depo). |
| 18 | 19 | The pill contains chemicals like the ones produced naturally in your body (called ‘hormones’) and must be taken daily. |
| 18 | 20 | There are two types of the pill: 1) the ‘secure pill’, made up of one chemical (hormone) and 2) the ‘control pill’, made up of two chemicals (hormones). |
| 19 | 21 | The control pill is usually taken every day for three weeks, followed by one week of non-active pills. After the week of non-active pills, a new control pill pack is started. |
| 19 | 22 | The secure pill must be taken every day within a certain time to work. When taking this pill, periods may not happen, which is something some women prefer. The secure pill is safe to take while breastfeeding. |
| 20 | 23 | To remember to take the pill, you could set an alarm on your phone or keep them near something that you do at the same time each day. |
| 21 | 24 | The pill can make periods regular and less severe. |
| 22 | 25 | Women can become pregnant soon after stopping the pill. |
| 23 | 26 | Most women do not gain or loose weight on the pill. |
| 24 | 27 | "The pill is easy to use and it made my skin clear from acne." |
| 25 | 28 | The intrauterine device ('loop', IUD) is very good at preventing pregnancy. |

| | | |
|---|---|---|
| 26 | 29 | The intrauterine device ('loop', IUD) is a small T-shaped piece of plastic and copper that is placed inside the uterus (womb) by a trained provider at a service. |
| 27 | 30 | The intrauterine device ('loop', IUD) can stay inside for 5-10 years, but it can be taken out at any time. It is possible to get pregnant shortly after it is removed. |
| 28 | 31 | While many women do not have any changes in bleeding with the intrauterine device ('loop', IUD), some will. This usually settles down and is not harmful. |
| 29 | 32 | The intrauterine device ('loop', IUD) is a safe method that doesn't cause, cancer, birth defects or store dirt. |
| 30 | 33 | Women can use the intrauterine device ('loop', IUD) if they have or have not had children. It is possible to become pregnant after it is removed. |
| 31 | 34 | One kind of intrauterine device ('loop', IUD) releases a hormone and can stay inside for 5 years. Women may not get a period while using it. |
| 32 | 35 | "After having the loop, I felt comfortable and free from worrying about getting pregnant." |
| 33 | 36 | The injection (Depo) also contains hormones and is very good at preventing pregnancy. It is given at a clinic every 13-14 weeks. |
| 34 | 37 | Some women have gradual weight gain on the injection. |
| 35 | 38 | Monthly periods may change or even stop with the injection. Some people like this. |
| 36 | 39 | Many women get pregnant soon after stopping the injection or missing a dose. In others, it can take a few months. |
| 36 | 40 | "The injection is affordable and available at clinics. I also find it very guaranteed in preventing pregnancy." |
| 37 | 41 | The implant (Jadelle), like the intrauterine device ('loop', IUD), is method that does not require the woman to do anything day to day. |
| 38 | 42 | The implant (Jadelle) is 2 small plastic flexible rods placed under the skin in the upper arm by a service provider. |
| 38 | 43 | The implant slowly releases a hormone and works for 3-5 years. Some women have irregular periods or no period (which some people like). |

| | | |
|---|---|---|
| 39 | 44 | It is possible to get pregnant shortly after the implant it is removed. |
| 39 | 45 | "I prefer the implant because it does not need a lot of follow up" |
| 40 | 46 | If you have sex without contraception, emergency contraception is available. It works best the sooner taken after sex. |
| 40 | 47 | The kind of emergency contraception available in Zimbabwe is a pill where the longest you can wait to take it after sex is 3 days. |
| 41 | 48 | Now you have information about the best methods available for preventing pregnancy: pills, the intrauterine device ('loop', IUD), implant (Jadelle) injection (Depo). |
| 42 | 49 | If used correctly, it is very unlikely that someone would get pregnant using pills, the intrauterine device ('loop', IUD), implant (Jadelle) injection (Depo). |
| 42 | 50 | For example, the pill must be taken on time & the injection must be given on time. |
| 44 | 51 | If you do not like the contraception you are using, choose another rather than stop to avoid unintended pregnancy. |
| 44 | 52 | There are many different options, so it is likely that you will find one that you like. |
| 46 | 53 | There are many myths about contraceptive methods. |
| 47 | 54 | Most men and women do not feel the intrauterine device ('loop', IUD). If you do, it can be can adjusted. |
| 48 | 55 | Most women do not have any weight changes on the pill. |
| 50 | 56 | You may have heard that contraception can make it impossible to become pregnant. This is a myth. It is possible to get pregnant after stopping contraception, regardless of how long it is used. |
| 51 | 57 | It is unlikely that Jadelle will move position and stop working. |
| 52 | 58 | If you do not like a method, you can try other methods to find the right method for you. |

| | | |
|---|---|---|
| 53 | 59 | Contraception is safe under medical supervision. |
| 54 | 60 | There are many options. Take some time to think and find the method most suitable for you. |
| 55 | 61 | Deciding about contraception with your partner can help you avoid an unintended pregnancy. |
| 55 | 62 | If you think it would help, you can look back at your messages together. |
| 56 | 63 | But it may not be possible to speak with your partner about contraception. |
| 56 | 64 | You may find out that you have different ideas about what you want regarding contraception. |
| 56 | 65 | If your partner disapproves, talk to them about why you believe that it's a good decision for you. |
| 57 | 66 | The intrauterine device ('loop', IUD) and implant are easy to keep private. |
| 58 | 67 | Providers help people of different lifestyles regarding contraception. |
| 59 | 68 | It's about your health and you have the right to choose what is right for you regardless of how others think and feel. |
| 60 | 69 | It is a provider’s job to keep your visit to the service confidential and they are required to do so. |
| 61 | 70 | You may wonder what your partner thinks about contraception. |
| 61 | 71 | You can only really know what they think by talking to them. |
| 61 | 72 | Making decisions together helps avoid unintended pregnancies. |
| 62 | 73 | “I started out by asking him how would he feel if we waited a bit before trying for a baby.” |

| | | |
|---|---|---|
| 64 | 74 | Using contraception means that you don't have to worry about unintended pregnancies. |
| 66 | 75 | Now that you have information about contraception, you can decide what’s best for you. |
| 68 | 76 | You know that hormonal contraception is safe. |
| 69 | 77 | You know where to get condoms and contraception and how to use them correctly. |
| 70 | 78 | You know that to avoid an unintended pregnancy, it is best to choose another method instead of stopping the current method. |
| 71 | 79 | Maybe you feel more confident talking to your partner about contraception. |
| 71 | 80 | Also, you may feel more comfortable talking to providers. |
| 71 | 81 | You are ready to decide for yourself. |
| 73 | 82 | Using contraception means that you don't have to worry if sex is unplanned. |
| 75 | 83 | Talking with your partner about contraception makes it more likely that you'll avoid unplanned pregnancy. |
| 77 | 84 | Knowing the benefits about contraception helps when talking to your partner about it. |
| 79 | 85 | Imagine different ways of starting the conversation with your partner. |
| 79 | 86 | Think about what you would say and picture yourself saying it. |
| 80 | 87 | Picture yourself asking what they think. |
| 81 | 88 | Picture yourself listening to them. |

| | | |
|---|---|---|
| 83 | 89 | "We were talking about kids and I asked her- 'when do you think is the right time to have children'?" |
| 84 | 90 | Every person has the right to go to a service and choose a method even though society may disapprove. |
| 85 | 91 | Using contraception means you decide when you want to become pregnant. |
| 86 | 92 | Contraception is also for people who have children. |
| 87 | 93 | Spacing births can improve the well-being of the baby and the whole family. |
| 87 | 94 | The intrauterine device ('loop', IUD) is a good method to use between pregnancies. It is safe while breastfeeding. |
| 88 | 95 | It is possible to get pregnant shortly after the intrauterine device ('loop', IUD) is removed. |
| 89 | 96 | Thinking about contraception is the first step towards making your own decision. |
| 90 | 97 | You have reached the end of the messages. We hope they have been helpful. |
