# Supplementary material for: Adapting an evidence-based contraceptive behavioural intervention delivered by mobile phone for young people in Zimbabwe
Source: BMC Health Serv Res. 2022 Jan 25;22:106. doi: 10.1186/s12913-022-07501-9 (PMC8789333; doi:10.1186/s12913-022-07501-9)
Supplement: Supplementary file 6 — Additional file 6. [file 12913_2022_7501_MOESM6_ESM.pdf]

Consolidated criteria for reporting qualitative studies (COREQ): 32-item checklist

Developed from:

Tong A, Sainsbury P, Craig J. Consolidated criteria for reporting qualitative research (COREQ): a 32-item checklist for interviews and focus groups. *International Journal for Quality in Health Care*. 2007. Volume 19, Number 6: pp. 349 – 357

No. Item	Guide questions/description	Section where reported
**Domain 1: Research team and reflexivity**		
Personal Characteristics		
1. Interviewer/facilitator	Which author/s conducted the interview or focus group?	Methods
2. Credentials	What were the researcher's credentials? E.g. PhD, MD	Methods
3. Occupation	What was their occupation at the time of the study?	Methods
4. Gender	Was the researcher male or female?	Methods
5. Experience and training	What experience or training did the researcher have?	Methods
Relationship with participants		
6. Relationship established	Was a relationship established prior to study commencement?	no, not reported
7. Participant knowledge of the interviewer	What did the participants know about the researcher? e.g. personal goals, reasons for doing the research	not reported
8. Interviewer characteristics	What characteristics were reported about the inter viewer/facilitator? e.g. Bias, assumptions, reasons and interests in the research topic	Methods
**Domain 2: study design**		
Theoretical framework		
9. Methodological orientation and Theory	What methodological orientation was stated to underpin the study? e.g. grounded theory, discourse analysis, ethnography, phenomenology, content analysis	Methods
Participant selection		
10. Sampling	How were participants selected? e.g. purposive, convenience, consecutive, snowball	Methods
11. Method of approach	How were participants approached? e.g. face-to-face, telephone, mail, email	Methods
12. Sample size	How many participants were in the study?	Results
13. Non-participation	How many people refused to participate or dropped out? Reasons?	not reported
Setting		
14. Setting of data collection	Where was the data collected? e.g. home, clinic, workplace	Methods and Results

15. Presence of non-participants	Was anyone else present besides the participants and researchers?	Results
16. Description of sample	What are the important characteristics of the sample? e.g. demographic data, date	Results
Data collection		
17. Interview guide	Were questions, prompts, guides provided by the authors? Was it pilot tested?	Methods
18. Repeat interviews	Were repeat inter views carried out? If yes, how many?	N/A
19. Audio/visual recording	Did the research use audio or visual recording to collect the data?	Methods
20. Field notes	Were field notes made during and/or after the inter view or focus group?	N/A
21. Duration	What was the duration of the inter views or focus group?	Methods
22. Data saturation	Was data saturation discussed?	no, not reported
23. Transcripts returned	Were transcripts returned to participants for comment and/or correction?	no, not reported
**Domain 3: analysis and findings**		
Data analysis		
24. Number of data coders	How many data coders coded the data?	Methods
25. Description of the coding tree	Did authors provide a description of the coding tree?	no, not reported
26. Derivation of themes	Were themes identified in advance or derived from the data?	Methods
27. Software	What software, if applicable, was used to manage the data?	Methods
28. Participant checking	Did participants provide feedback on the findings?	no, not reported
Reporting		
29. Quotations presented	Were participant quotations presented to illustrate the themes/findings? Was each quotation identified? e.g. participant number	Results
30. Data and findings consistent	Was there consistency between the data presented and the findings?	Discussion
31. Clarity of major themes	Were major themes clearly presented in the findings?	Results
32. Clarity of minor themes	Is there a description of diverse cases or discussion of minor themes?	Discussion
